# Precision Rehabilitation Through Mathematical Modeling: A New Frontier in Medicine

**DOI:** 10.7759/cureus.86965

**Published:** 2025-06-29

**Authors:** Raktim Swarnakar

**Affiliations:** 1 Physical Medicine and Rehabilitation, National Cancer Institute, Jhajjar Campus, All India Institute of Medical Sciences, New Delhi, IND

**Keywords:** individualized therapy, mathematical modeling, outcome measurements, precision therapy, rehabilitation

## Abstract

Rehabilitation medicine is undergoing a significant transformation with the integration of precision-based approaches grounded in mathematical modeling. Traditional rehabilitation protocols, often generalized and uniform, fail to capture the diverse recovery patterns seen in patients with neurological and musculoskeletal injuries such as stroke, spinal cord injury, or traumatic brain injury. To address this variability, mathematical modeling can be used to predict functional recovery over time through a differential equation that incorporates therapy intensity, baseline function, individual recovery potential, and the natural recovery plateau. Key parameters include functional ability at time *t* (F(t)), baseline function (B), therapy intensity (T), recovery potential (R), therapy efficacy (α), and recovery plateau rate (λ). These variables can be estimated using clinical data, validated prediction models, and modern machine learning algorithms trained on large datasets. Such models enable clinicians to forecast outcomes, individualize treatment plans, compare intervention strategies, and set realistic recovery goals. Rather than replacing clinical expertise, mathematical modeling enhances it by providing a quantitative framework to guide decision-making. As healthcare continues to evolve, these models can form the basis for real-time, adaptive rehabilitation strategies integrated with electronic health records and wearable technologies. From now on, precision rehabilitation supported by mathematical modeling offers a practical and evidence-based path toward more personalized and effective patient care.

## Editorial

Rehabilitation medicine is entering an era of profound transformation. Once reliant on generalized therapy protocols, the field is now shifting toward precision rehabilitation, an approach that recognizes the unique recovery potential of each patient. At the heart of this evolution lies mathematical modeling, a method that translates complex clinical realities into structured, data-driven frameworks. By bridging the gap between empirical care and predictive science, mathematical models offer a new frontier for truly individualized rehabilitation [1].

Functional recovery following neurological or musculoskeletal injury varies dramatically between patients. Stroke, spinal cord injury, and traumatic brain injury all result in a wide spectrum of outcomes shaped by age, comorbidities, baseline impairments, therapy intensity, and neuroplastic potential. Conventional rehabilitation strategies often fail to accommodate this diversity, potentially leading to under- or over-treatment. Mathematical models provide a powerful means of tailoring rehabilitation intensity and duration to each patient’s unique trajectory. A previous study demonstrated the use of mathematical modeling to represent post-surgical knee extension recovery, showing how functional improvement can be tracked over time using empirical data [2].

To predict or describe functional recovery over time, allowing clinicians to anticipate outcomes and optimize care strategies, the rate of functional improvement can be modeled as a first-order differential equation: \[ \frac{dF(t)}{dt} = \alpha \cdot T(t) \cdot R - \lambda \cdot \left( F(t) - B \right) \]. Additionally, the functional ability at time t can be calculated as: \[ F(t) = B + \frac{\alpha \cdot T_0 \cdot R}{\lambda} \left( 1 - e^{-\lambda t} \right) \]

In this model, the derivative dF(t)/dt represents the rate of functional recovery at time t. The term α·T(t)·R reflects therapy-driven improvement, scaled by the patient’s individual recovery potential, while the term λ·(F(t)−B) represents the diminishing rate of improvement as the patient approaches their functional ceiling or a natural plateau. Assuming therapy intensity remains constant over time (T(t) = T₀), the solution to this differential equation yields an exponential recovery function, as depicted in the model's analytical expression (second equation).

In this formulation, F(t) denotes the functional ability at time t, such as measured by the Functional Independence Measure (FIM) or the Barthel Index. B represents the baseline function at admission. The recovery potential, R, is based on clinical factors such as age, comorbidities, and severity of injury, and may be normalized on a scale from 0 to 1 using a heuristic approach. Several validated prediction models in stroke and neurorehabilitation already utilize multivariable inputs, including age, neurological status, and comorbid conditions, to predict recovery trajectories [3]. Modern machine learning models trained on large datasets can also be used to generate recovery potential scores between 0 and 1, thereby providing a data-driven estimation of R. The term T(t) indicates therapy intensity at time t, typically measured in hours per day or categorized by type and complexity of therapy. T(0) denotes the therapy intensity at time zero (initial), while e represents the exponential constant known as Euler's number.

Alpha (α) is defined as the therapy effectiveness coefficient. It represents the efficiency of functional improvement per unit of therapy and is best estimated empirically using clinical data. This requires analyzing functional gains in relation to therapy intensity while controlling for other covariates such as R and λ. In this way, alpha serves as a quantitative abstraction of dose-response relationships observed in rehabilitation trials [4]. Lambda (λ), the plateau or decay factor, represents the rate at which recovery slows over time. It captures the phenomenon that functional improvement is typically most rapid in the early phase and gradually tapers [5]. This parameter should be estimated from longitudinal functional outcome data using statistical curve-fitting techniques.

This mathematical framework has practical clinical utility. For example, consider a 65-year-old stroke patient with moderate baseline impairments (B = 40 on the FIM), a recovery potential score of R = 0.6, and receiving 2 hours of therapy daily (T₀ = 2). Using empirically derived values for α and λ, the model can estimate the patient's expected functional improvement over time and predict whether their FIM score will reach a target discharge level (e.g., F = 80) within a given inpatient stay. If the model projects suboptimal recovery under current conditions, therapy intensity or duration can be adjusted accordingly to optimize outcomes. It can be used to predict discharge function, compare different rehabilitation strategies, such as high versus low therapy intensity, and individualize treatment plans and therapy duration. Mathematical modeling does not replace clinical judgment; rather, it sharpens it. These models help physiatrists and therapists forecast recovery pathways with greater accuracy, align rehabilitation resources with clinical needs, and set patient-specific, data-informed goals.

One potential challenge to implementing this framework is the availability and quality of data needed to reliably estimate model parameters such as α, λ, and R. Inconsistent documentation, variability in therapy reporting, and limited access to longitudinal functional outcomes can hinder model accuracy. Addressing this requires improved data capture through standardized assessments, integration of electronic health records, and broader adoption of digital health tools.

Furthermore, the presented mathematical model can be extended to incorporate dynamic physiological changes by allowing key parameters, such as recovery potential (R) and therapy effectiveness (α), to vary over time rather than remain fixed. By integrating longitudinal data from wearable sensors, clinical assessments, or biomarkers, these parameters can be updated continuously, enabling the model to reflect evolving patient status and responsiveness to therapy. This adaptive framework supports real-time adjustment of rehabilitation intensity and duration, ensuring therapy remains aligned with the patient’s current biological potential and optimizing recovery trajectories. It is also important to acknowledge that parameters such as therapy responsiveness (α), recovery potential (R), and decay rate (λ) are often individualized, time-variant, and influenced by unmeasured psychosocial or biological factors, adding layers of complexity to their estimation and interpretation in real-world rehabilitation settings.

As rehabilitation integrates more advanced technology, including wearable sensors, electronic health records, and patient-reported outcome tools, the potential for adaptive, real-time modeling becomes increasingly feasible. This will further personalize rehabilitation by dynamically adjusting therapy in response to patient progress.

As healthcare moves toward greater personalization, precision rehabilitation through mathematical modeling offers a logical and evidence-based approach to treating the whole patient. It ensures that each hour of therapy is purposefully aligned with the patient’s biological potential and functional needs. Rehabilitation medicine must embrace this new frontier, not only to enhance recovery outcomes but to fundamentally redefine how we understand, monitor, and guide the process of healing itself.

In conclusion, mathematical modeling represents a transformative step toward precision rehabilitation, enabling clinicians to tailor therapy to each patient’s unique recovery profile. By converting clinical variability into structured, predictive insights, these models support more accurate goal-setting, resource planning, and outcome forecasting. As technology advances and real-time data integration becomes routine, adaptive modeling will allow rehabilitation strategies to evolve dynamically with patient progress. Far from replacing clinical judgment, these tools enhance it, ensuring that every therapeutic decision aligns with biological potential and functional needs. This approach not only improves outcomes but also redefines how recovery is understood and guided in modern rehabilitation medicine.
